# A quantum mechanical computational method for modeling electrostatic and solvation effects of protein

**DOI:** 10.1038/s41598-018-23783-8

**Published:** 2018-04-03

**Authors:** Xianwei Wang, Yang Li, Ya Gao, Zejin Yang, Chenhui Lu, Tong Zhu

**Affiliations:** 1 College of Science, Zhejiang University of Technology, Hangzhou, Zhejiang, 310023 China; 2 School of Information Science and Engineering, Shandong Agricultural University, Taian, 271018 China; 3 College of Fundamental Studies, Shanghai University of Engineering Science, Shanghai, 201620 China; 4 College of Mechanical Engineering, Shanghai University of Engineering Science, Shanghai, 201620 China; 5 College of Chemistry and Molecular Engineering, East China Normal University, Shanghai, 200062 China; 6 YU-ECNU Center for Computational Chemistry at NYU Shanghai, Shanghai, 200062 China

## Abstract

An efficient computational approach for modeling protein electrostatic is developed according to static point-charge model distributions based on the linear-scaling EE-GMFCC (electrostatically embedded generalized molecular fractionation with conjugate caps) quantum mechanical (QM) method. In this approach, the Electrostatic-Potential atomic charges are obtained from *ab initio* calculation of protein, both polarization and charge transfer effect are taken into consideration. This approach shows a significant improvement in the description of electrostatic potential and solvation energy of proteins comparing with current popular molecular mechanics (MM) force fields. Therefore, it has gorgeous prospect in many applications, including accurate calculations of electric field or vibrational Stark spectroscopy in proteins and predicting protein-ligand binding affinity. It can also be applied in QM/MM calculations or electronic embedding method of ONIOM to provide a better electrostatic environment.

## Introduction

Electrostatic interaction plays a central role in many molecular processes in biological molecules^1–5^, including protein folding^6^, protein-ligand binding^7,8^, protein-protein interaction^9^, electron transfer^10^, enzyme reaction^11,12^, ion channels^13,14^, etc. The molecular electrostatic potential (MEP) has been widely used to characterize inter-and intramolecular electrostatic interactions. A great deal of progress has been made over the past decades in the development of rigorous and practical methods for accurate description of the MEP of proteins^9,15–22^.

The point charge model adopted in standard MM force field is widely used in simulations of electrostatic properties of proteins, including introducing the solvation effect in simulations of proteins by incorporating with implicit solvent model^23,24^, modeling electrostatic potential of protein^9,25^, electric field at the active site of protease^26–28^ and vibrational Stark spectroscopy in protein^29,30^, providing the electrostatic environment as background charges in QM/MM calculations^31–33^ or electronic embedding method of ONIOM^34–36^, etc. Although MM force fields have made great success in studying thermodynamic and kinetic properties of biomolecules, there are fundamental limitations in their applications. The atomic charges of each kind of amino acid in standard MM fore field are obtained from *ab initio* QM calculations of small model systems^21,22,37–39^. For instance, the atomic charges used in Amber99SB force field are obtained from gas phase HF/6-31 G* QM calculations of small peptides^37^. The atomic charges are static and fixed, and therefore do not contain quantum mechanical information (polarization and charge transfer effect) of a particular protein structure. It is well known that the local electrostatic environment inside a folded protein is inhomogeneous due to the specific organization of charged and polar groups. The electron-density distribution of each amino acid in a particular protein electrostatic environment is specific due to polarization and charge transfer effect. Many previous works have demonstrated that the simulations based on these standard MM force fields are incapable of giving a quantitative comparison and interpretation of experimental observables^9,21,22,26–28^.

To overcome the fundamental deficiency of the fixed charge model used in the standard MM force fields and describe electrostatic environment of proteins accurately, many efforts have been made to develop a new generation of polarizable force field^16,40^. Introducing excess parameterizations (such as induced dipole, etc.) in current standard MM force fields is a common method^41–43^. However, parametrization often makes the applications of the polarizable force field much more complicated than that of standard MM force field regarding the accuracy and validity of the underlying theoretical models used to derive polarizable force field remain. To accurately account for polarization and charge transfer effects without introducing excess parameterizations, it is usually necessary to adopt first-principles electronic structure methods. However, it is still not practical to apply standard quantum mechanical methods for describing the full inhomogeneous electrostatic environment of the proteins^44^. The major limitation of QM methods is the scaling problem. The Hartree-Fock (HF) and density functional theory (DFT) scales as O(*N*^3^) (*N* denotes the size of the system). The scaling of post-HF methods is O(*N*^5^) for second-order Moller-Plesset perturbation theory (MP2) and O(*N*^6^) for the coupled-cluster (CC) method that includes single and double excitations (CCSD), respectively.

To overcome the scaling limitation of the applications of rigorous electronic-structure methods in large systems, various linear-scaling methods have been developed over the past decade^45^. Among the existing linear-scaling QM approaches, the fragmentation approach is one of the highly efficient and powerful methods. The fragmentation approach is on the basis of the “chemical locality” of most large molecular systems, which assumes that the local region of the large system is only weakly influenced by the atoms that are far away from this region. Based on this chemical intuition, the system is divided into many individual subsystems (fragments) and subsequently the properties of the whole system can be obtained by taking a linear combination of the properties of these fragments. Over the past decade, many fragmentation QM methods have been proposed^46,47^, including the fragment molecular orbital (FMO) method^48^, the systematic fragmentation method (SFM)^49^, the molecular tailoring approach (MTA)^50^, the molecular fractionation with conjugate caps (MFCC) method^21,51^, the adjustable density matrix assembler (ADMA) method^52^, the electrostatically embedded many-body (EE-MB) expansion approach^53^, the explicit polarizatioin (X-Pol) potential^54^.

With the goal of obtaining more accurate electronic structure properties of proteins, we have proposed a linear-scaling QM method termed EE-GMFCC (electrostatically embedded generalized molecular fractionation with conjugate caps method)^55^. In the calculations of total energies of proteins, the EE-GMFCC shows only a few kcal/mol deviation from the corresponding full system (FS) results at the levels of the HF, DFT and MP2 method. With respect to expensive conventional QM methods, the EE-GMFCC greatly reduces the computational cost of QM calculations, extending the applicability of the rigorous QM methods to proteins with any number of atoms (up to thousands of atoms or more). The EE-GMFCC method is linear-scaling with a low prefactor. The relative independence of the QM calculations of the fragments in the EE-GMFCC method makes it suitable for implementation of parallelization. The applications of the EE-GMFCC method have been extended to perform structural optimization of proteins^56^ and molecular dynamics simulations with high level *ab initio* electronic structure theories^57^.

In this paper, based on accurate electronic structure calculations of proteins with the EE-GMFCC method, an electrostatic potential (ESP) atomic charges computational approach was developed and used in the calculations of electrostatic potentials and the solvation energies of proteins. By comparison with the results of FS QM calculations, the capabilities of the new charge model are demonstrated and new physical insights obtained from accurate description of protein electrostatic properties are discussed.

## Method

### EE-GMFCC method

**Figure 1:**
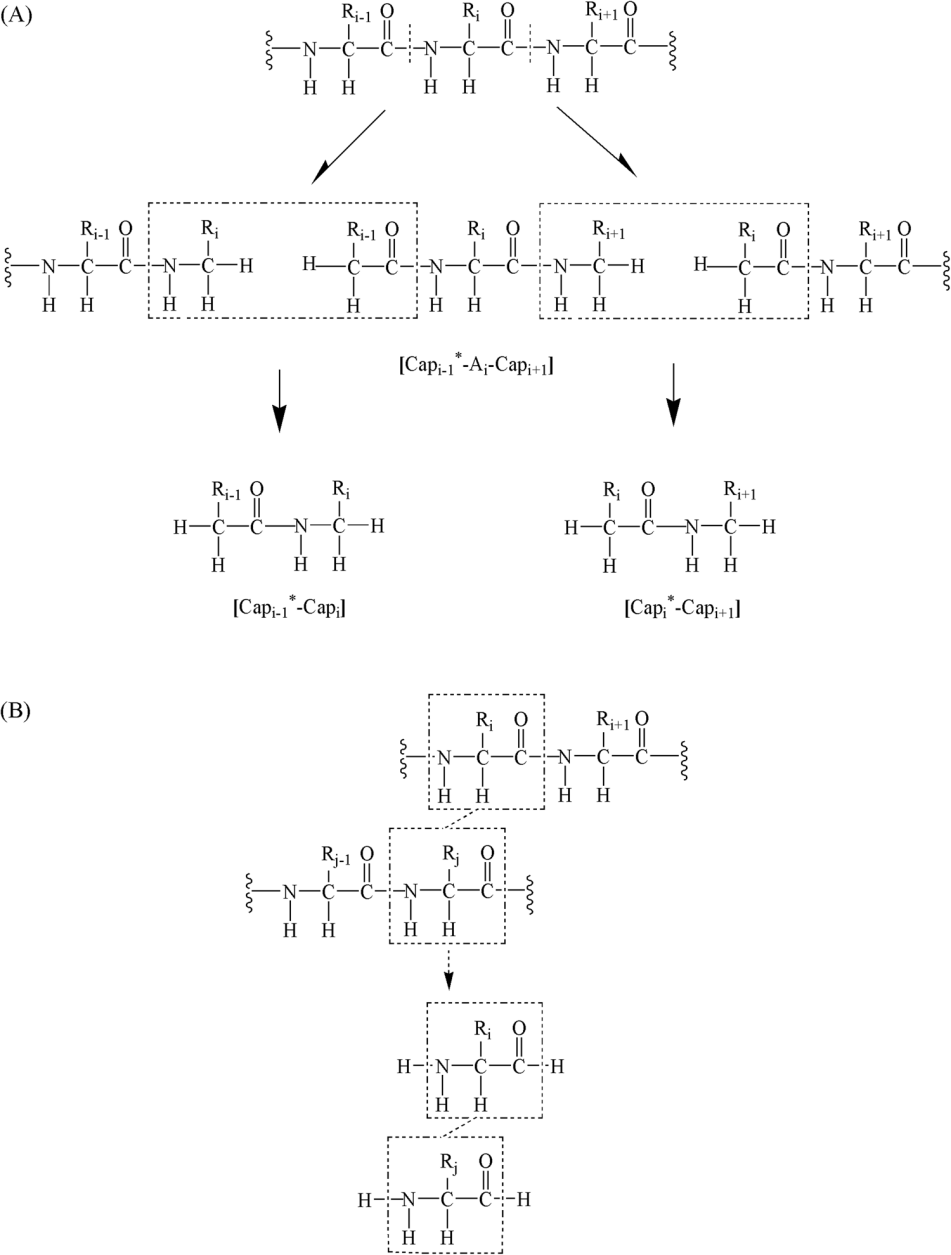
(**A**) The cutting scheme of EE-GMFCC method. The ${{\rm{Cap}}}_{i+1}$ and its conjugate ${{\rm{Cap}}}_{i}^{\ast }$ are used for capping the fragments, where *i* denotes the index of the *i*th amino acid in the given protein. The i*th* fragment is defned as ${{\rm{Cap}}}_{i-1}^{\ast }-{{\rm{A}}}_{i}-{{\rm{Cap}}}_{i+1}$ and the i*th* concap is defned as ${{\rm{Cap}}}_{i}^{\ast }-{{\rm{Cap}}}_{i+1}$. (**B**) The cutting scheme of the generalized concap (Gconcap) and the atomic structure of the Gconcap. (See ref.^55^ for more details).

The EE-GMFCC method is initially developed for calculating protein energy (see refs^51,55^. for more details). Here, we just give a brief review. In the framework of EE-GMFCC method, a protein is decomposed into a number of individual fragments in the unit of amino acid by cutting through the peptide bond as illustrated in Fig. 1. A pair of conjugate caps (concaps) is inserted at the cutting location to mimic the local chemical environment of the original protein to the cutoff fragments (see Fig. 1A). Two-body terms for the interaction energy between non-neighboring residues that are close in space are also introduced to capture short-range quantum effect (see Fig. 1B). All the fragment calculations are embedded in the electrostatic field of the point charges representing the remaining atoms in the protein. The point charge model is taken from the Amber94 force field. Hydrogen atoms are used to saturate the dangling bonds. The total energy of a protein (with *N* amino acids) using the EE-GMFCC method can be expressed as

1 $$E=\sum _{i=2}^{N-1}\tilde{E}({{\rm{Cap}}}_{i-1}^{\ast }{{\rm{A}}}_{i}{{\rm{Cap}}}_{i+1})-\sum _{i=2}^{N-2}\tilde{E}({{\rm{Cap}}}_{i}^{\ast }{{\rm{Cap}}}_{i+1})-\sum _{\begin{array}{c}i,j > i+2\\ |{R}_{i}-{R}_{j}|\le \lambda \end{array}}({\tilde{E}}_{ij}-{\tilde{E}}_{i}-{\tilde{E}}_{j})-{E}_{{\rm{DC}}}$$

where *i* and *j* represent the residue number and $\tilde{E}$ denotes the sum of the self-energy of the fragment and the interaction energy between the fragment and background charges of the remaining system. ${\tilde{E}}_{ij}-{\tilde{E}}_{i}-{\tilde{E}}_{j}$ represents the two-body QM interaction energy between residues *i* and *j* whose closest distance is less than a predefined threshold $\,{\rm{\lambda }}$. ${E}_{{\rm{DC}}}$ is the interaction energy doubly counted in the first three terms of Eq. (1) and is approximated by the pairwise charge-charge interactions. The complete definition of the $\,{E}_{{\rm{DC}}}$ can be found in refs^51,55^.

### Charge determination

To reproduce MEP as well as possible, the ESP fitting method is employed to determine the atomic charges. Based on the computational scheme of the EE-GMFCC method, the atomic charge of atom *k* in a protein can be obtained by the following equation:

2 $${q}_{k}=\sum _{i=2}^{N-1}{q}_{k}({{\rm{Cap}}}_{i-1}^{\ast }{{\rm{A}}}_{i}{{\rm{Cap}}}_{i+1})-\sum _{i=2}^{N-2}{q}_{k}({{\rm{Cap}}}_{i}^{\ast }{{\rm{Cap}}}_{i+1})-\sum _{\begin{array}{c}i,j > i+2\\ |{R}_{i}-{R}_{j}|\le \lambda \end{array}}({q}_{k}({{\rm{A}}}_{i}{{\rm{A}}}_{j})-{q}_{k}({{\rm{A}}}_{i})-{q}_{k}({{\rm{A}}}_{j}))$$

where $\,{q}_{k}({{\rm{Cap}}}_{i-1}^{\ast }{{\rm{A}}}_{i}{{\rm{Cap}}}_{i+1})$ and ${q}_{k}({{\rm{Cap}}}_{i}^{\ast }{{\rm{Cap}}}_{i+1})$ denote the ESP charge of atom *k* obtained from the quantum mechanical calculations of the fragment ${{\rm{Cap}}}_{i-1}^{\ast }{{\rm{A}}}_{i}{{\rm{Cap}}}_{i+1}$ and concap ${{\rm{Cap}}}_{i}^{\ast }{{\rm{Cap}}}_{i+1}$ respectively. Since atom *k* may be assigned to different fragments (there is overlap of neighboring fragments, see Fig. 1) and concaps, ${q}_{k}$ would be counted more than once after sum calculation of the first term of Eq. (2), while the double counting can be just deducted by subtracting the atom *k*^,^s charge in concaps. To avoid introducing unnatural excess charge in the process of charge fitting, the charges of link-atoms (hydrogen atom) of concap are constrained to have the same value with that of corresponding hydrogen atom in the fragment with penalty function in the RESP fitting, e.g., the charge of the hydrogen atom using as a link-atom of ${{\rm{Cap}}}_{i+1}$ in the concap ${{\rm{Cap}}}_{i}^{\ast }{{\rm{Cap}}}_{i+1}$ is constrained to have the same value with the link-atom of ${{\rm{Cap}}}_{i+1}$ in the fragment ${{\rm{Cap}}}_{i-1}^{\ast }{{\rm{A}}}_{i}{{\rm{Cap}}}_{i+1}$. This is essential and makes sense, because the local chemical environment of the corresponding hydrogen atoms in the fragment and concap is similar. The third term in Eq. (2) is used to capture quantum-mechanical two-body effect between nonsequentially connected residues that are spatially close.

The validity of the obtained charges from the EE-GMFCC method (termed EE-GMFCC-CHG) is tested with two protein systems (PDB id: 1BHI and 2KCF) using HF and DFT (M06-2×) methods with 6-31 G* basis set. The geometries of the two proteins were optimized with Amber99SB^38^ force field using Sander module of the Amber program^58^ in order to remove bad contacts prior to subsequent *ab initio* calculations. The calculated electrostatic potential using the EE-GMFCC-CHG were compared to that of the FS QM calculations. All *ab initio* calculations were performed using the Gaussian 09 program^59^.

### Solvent effect

In the continuum-solvent model, the solvent is represented as a continuous polarizable medium with dielectric constant ${\rm{\varepsilon }}$ and solute (protein) is encapsulated in a cavity with charge density ${\rm{\rho }}({\bf{r}})$ embedded in the medium. The solute polarizes the surrounding dielectric medium and creates a reaction potential which acts back to polarize the solute until equilibrium is reached. According to the classical electrostatic theory, the reaction potential acting on the solute can be effectively represented by that of induced charges on the surface of the cavity. In the Polarized continuum model (PCM), the solvation effect is modeled by discretizing the induced charges on the cavity surface and iteratively solving the quantum chemistry equation for the solute in the field of surface charges^60^. The PCM is a popular continuum-solvent method for incorporation of solvent effect in quantum mechanical calculations of small molecules. However, although the PCM method was generalized to model the solvation effect of large proteins based on linear-scaling quantum mechanical methods^61–63^, it still has limitations, e.g., many discrete surface charges will be required in the PCM which makes the solution of linear equation difficult computationally and the effect of ion concentrations is not included in the PCM method. In this work, by combining with continuum-solvent model based on the Poisson-Boltzmann (PB) equation, the EE-GMFCC-CHG will be used to model the electrostatic solvation effects of large proteins. The PB method has two advantages relative to the PCM method in modeling the solvation effects of large proteins. (1) The induced charges are obtained by numerically solving the PB equation which avoids the solution of large linear equations and improves the computation speed. (2) The PB equation incorporates the effect of ion concentrations which gives a better description of the real-environment of proteins.

In combinatorial point-charge fitting approach of EE-GMFCC-CHG and PB equation, partial charges of atoms of the protein generated from the EE-GMFCC quantum mechanical calculations were passed to the PB solver Delphi^24^ to derive the induced surface charges on the dielectric boundary. The dielectric solute/solvent boundary was defined by Amber van der Waals radii for protein molecule with a probe radius of 1.4 Å. The solvent and internal dielectric constants are set to 80 and unity respectively. The obtained surface charges are added to the background charges to the next EE-GMFCC quantum-chemical calculations to generated new partial charges of the protein. The partial charges of the protein and induced surface charges generated by Delphi polarize each other until converge was reached. This process was iterated until the corrected reaction field energy calculated with Delphi converged and its variations were smaller than a certain criterion. Usually, the criterion was reached within five iterations.

The capability of EE-GMFCC-CHG in predicting the relative electrostatic solvation energy was demonstrated using 20 different conformations of a small protein (PDB id: 2I9M) generated from a 2 ns MD simulation. The MD simulation was performed with Amber99SB force field^37^ and TIP3P water model to handle the protein and solvent (the specific process of the MD simulation is the same as that in ref.^63^). The conformations were selected from the trajectory every 100 ps. MD simulations were performed with the Amber 12 program^58^.

## Results and Discussion

### EE-GMFCC-CHG Reproduces *ab initio* Electrostatic Potential

Correct description of the electrostatic is vital in accurate prediction of molecular interactions in biological systems. Although the standard non-polarizable force fields (such as Amber, CHARMM, etc.) have achieved successes in simulating many of the macroscopic properties of proteins, it is expected to have difficulties in giving accurate prediction of properties that are more sensitive to the local electrostatic environment. This originates from the fact that the point-charge model of standard force fields is mean-field-like and it does not contain protein-specific quantum mechanical information such as polarization effect, charge transfer effect, etc. The EE-GMFCC-CHG derives from the quantum-chemical calculations that are performed using the EE-GMFCC fragmentation methods. To account for the protein polarization, the calculations of all molecular fragments are in the field created by point charges of the remaining system. By treatment of nonsequentially connected residues that are spatially close with generalized concaps (Gconcaps), polarization and charge transfer effect are accurately included. EE-GMFCC-CHG should reproduce *ab initio* electrostatics of proteins better than standard MM force field.

**Figure 2:**
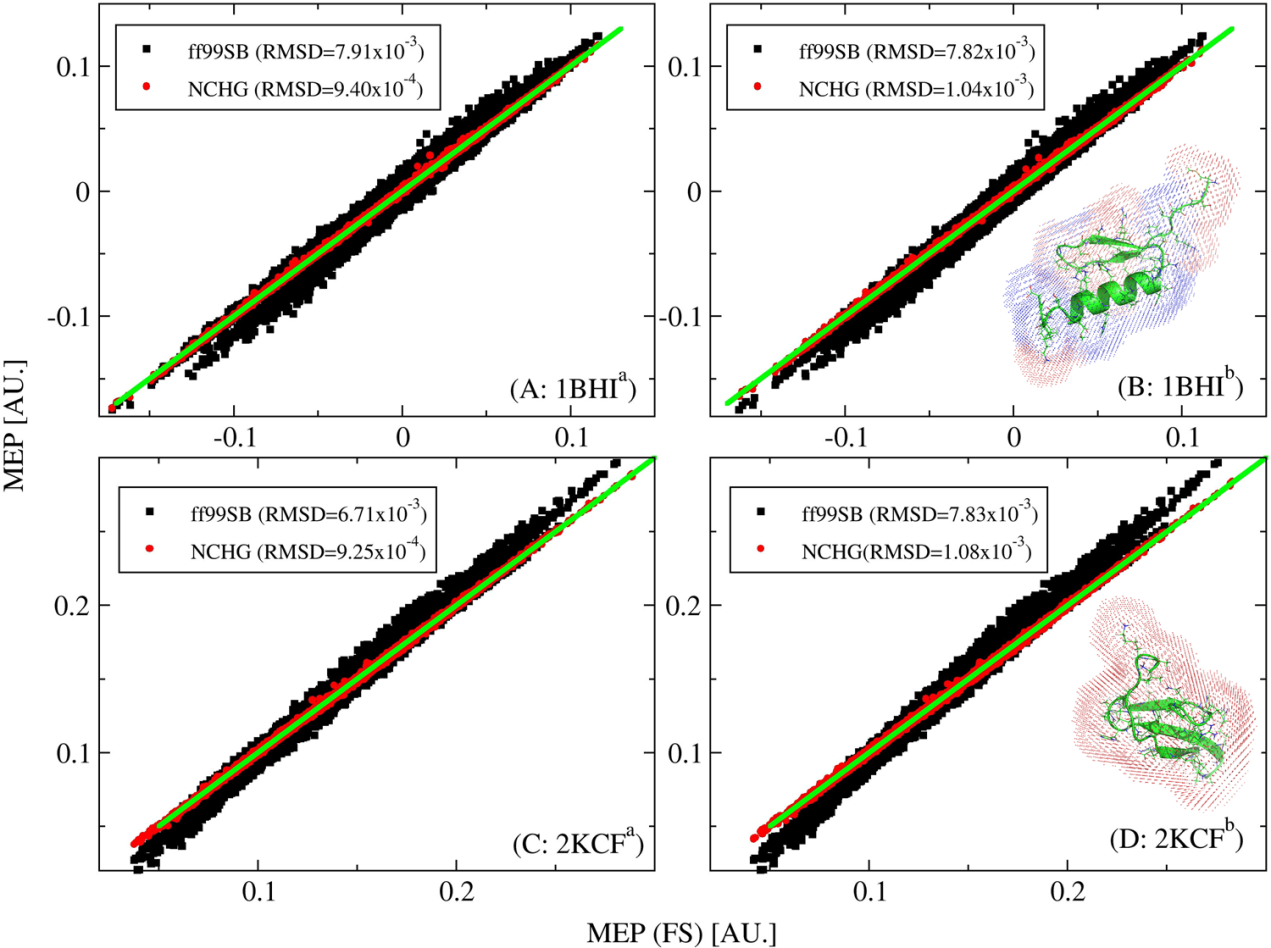
Correlations of MEP (in atomic units) between FS QM calculations and ff99SB (black), EE-GMFCC-CHG (marked as NCHG, red), respectively, for two small globular proteins (PDB id: 1BHI (top) and 2KCF (bottom)). The representative three-dimensional protein structures of two small globular proteins and the grid point where electrostatic potentials are calculated are also shown in panel B and D. ^a^The corresponding QM calculations are performed at HF/6-31 G* level. ^b^The corresponding QM calculations are performed at M06-2×/6-31 G* level.

We have calculated the electrostatic potential of the two real three-dimensional proteins (PDB id: 1BHI and 2KCF) that contain 591 and 571 atoms respectively. The secondary structure of the protein 1BHI is a mixture of ${\rm{\alpha }}$-helix and ${\rm{\beta }}$-sheet, while 2KCF contains ${\rm{\beta }}$-sheet primarily. Since the electrostatic potential near a molecule are location-dependent, they will become very large near the nuclei in many cases. So the electrostatic potentials at grid points which are from 2.5 to 4.5 Å away from the closest atom in the protein were calculated. The three-dimensional protein structures of 1BHI and 2KCF and the grid points are shown in Fig. 2. The calculated molecular electrostatic potential with the FS QM method is chosen as benchmark and are compared to the corresponding results obtained according to the EE-GMFCC-CHG and Amber99SB force field approach.

The benchmark *ab initio* calculations are performed at HF/6-31 G* and M06-2×/6-31 G* level of theory respectively. The correlations between the benchmark QM calculations and the EE-GMFCC-CHG and Amber99SB force field approach are shown in Fig. 2. From panels (A) and (C) in Fig. 2, one can see that the calculated electrostatic potential using Amber99SB force field shows large root-mean-square deviation (RMSD) of MEP from FS calculations at the HF/6-31 G* level. While the obtained MEPs based on the EE-GMFCC-CHG are in excellent agreement with the results from FS calculations. There is an order of magnitude improvement in RMSD for the EE-GMFCC-CHG method as compared to Amber99SB force field. These results demonstrate that although the charge model used in the Amber99SB force field was obtained by fitting the gas-phase electrostatic potential of small peptides calculated at the HF/6-31 G* level, it can still not reproduce accurate electrostatic properties of protein due to lack of the polarization and charge transfer effect. Because of introducing polarization and charge transfer effect (including the charge transfer effect of sequentially and nonsequentially connected residues) by rigorous *ab initio* quantum chemistry calculations in the charge fitting process of EE-GMFCC-CHG, EE-GMFCC-CHG shows a significant improvement in describing the electrostatic environment with respect to Amber99SB force field.

Since the electron correlation is not included in the HF method, the calculated molecular dipole moment with the HF method is usually overestimated. The electrostatic properties of proteins predicted using the HF method are not accurate enough. While the DFT method such as M06-2× functional can give much better prediction in electrostatics of proteins. To test the accuracy of the EE-GMFCC-CHG, the correlation between the calculated MEP based on the EE-GMFCC-CHG and FS QM calculations for the two proteins (1BHI and 2KCF) at the M06-2×/6-31 G* level are plotted in panels (B) and (D) of Fig. 2. Similar to the results based on the HF method, the calculated MEP of the two proteins base on the EE-GMFCC-CHG shows very small RMSD from the corresponding FS QM calculations and also shows about an order of magnitude improvement in RMSD as compared to the results of Amber99SB force field.

**Figure 3:**
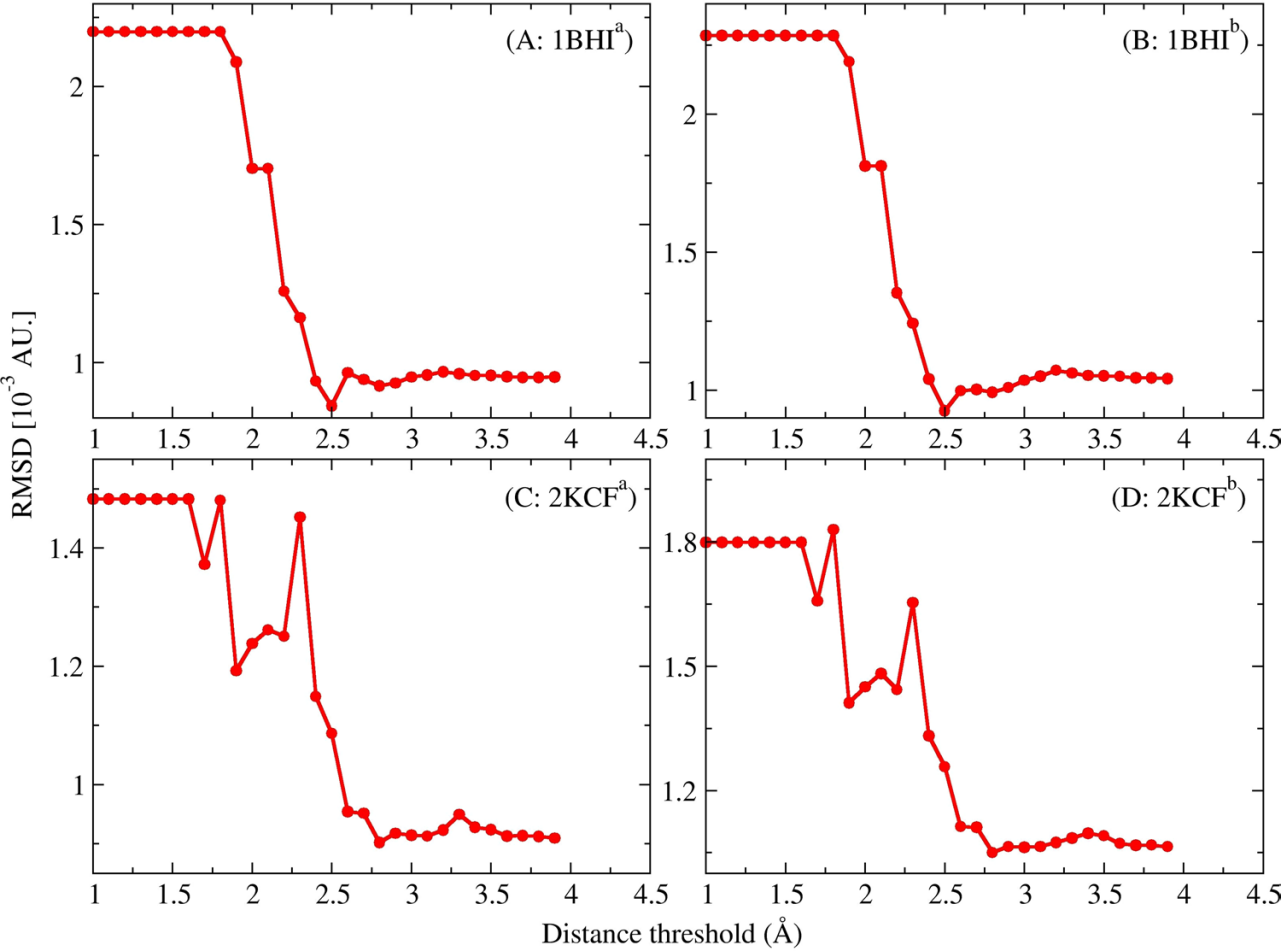
The RMSD of calculated electrostatic potential as a function of the distance threshold for the two proteins at ^a^HF/6-31 G* and ^b^M06-2×/6-31 G* level. The calculated electrostatic potential from the full system calculation is regarded as the reference.

To investigate the role in reproducing the *ab initio* QM electrostatic potential of introducing the two-body effect (quantum mechanical polarization and charge transfer effect) for nonsequentially connected residues that are spatially close, we plot the evolution of RMSD of MEP over the distance threshold ${\rm{\lambda }}$ as reference to the FS QM results in Fig. 3. Figure 3 demonstrates that the RMSD are all close to convergence at $\,{\rm{\lambda }}$ = 4 Å. The closest non-neighboring fragment appears when ${\rm{\lambda }}\,\,$is about 1.7–1.9 Å for the two globular proteins. The RMSDs of MEP based on the EE-GMFCC-CHG at the HF/6-31 G* level are about 2.2 × 10^−3^ au. and 1.5 × 10^−3^ au. (see panel (A) and (C) in Fig. 3 when the distance threshold $\,{\rm{\lambda }}\,\,$is less than 1.7 Å) for 1BHI and 2KCF in the case that the two-body effect is not introduced. Compared with Amber99SB force field, the error is reduced by about 5.7 × 10^−3^ au. (about 72% relative to the result based on Amber99SB force field) and 5.2 × 10^−3^ au. (about 78% relative to the result based on Amber99SB force field) respectively. The RMSDs of MEP using the EE-GMFCC-CHG without introducing two-body effect are also reduced by about 70% for 1BHI and 76% for 2KCH at M06-2×/6-31 G* level. The results indicate that the polarization effect and charge transfer from neighboring residues play a significant role in accurate description of protein electrostatic. From Fig. 3, one can see that the introduction of the two-body effect can reduce the RMSD of MEP obviously. The RMSDs are reduced to 9.4 × 10^−3^ au. (HF) and 1.04 × 10^−3^ au. (M06-2×) for 1BHI and 9.25 × 10^−3^ au. (HF) and 1.08 × 10^−3^ au. (M06-2×) for 2KCF when $\,{\rm{\lambda }}\,\,$is set to 4.0 Å which indicates that the two-body QM correction of vicinal non-neighboring residues is crucial to reproducing accurate electrostatic properties of proteins. It is worth noting that the RMSDs are markedly reduced when distance threshold ${\rm{\lambda }}\,\,$is increased from 1.7 to 2.5 Å. The RMSDs are almost flat when $\,{\rm{\lambda }}\,\,$is increased from 2.7 to 4.0 Å which suggests that the quantum mechanical effect of non-neighboring residues is local and a smaller distance threshold $\,{\rm{\lambda }}\,$(such as 2.7 Å) is still appropriate for the two globular proteins. Adoption of an appropriate distance threshold $\,{\rm{\lambda }}\,\,$for introducing two-body effect in fitting EE-GMFCC-CHG is essential for reducing computational cost.

### Electrostatic solvation energy calculation using the EE-GMFCC-CHG

**Figure 4:**
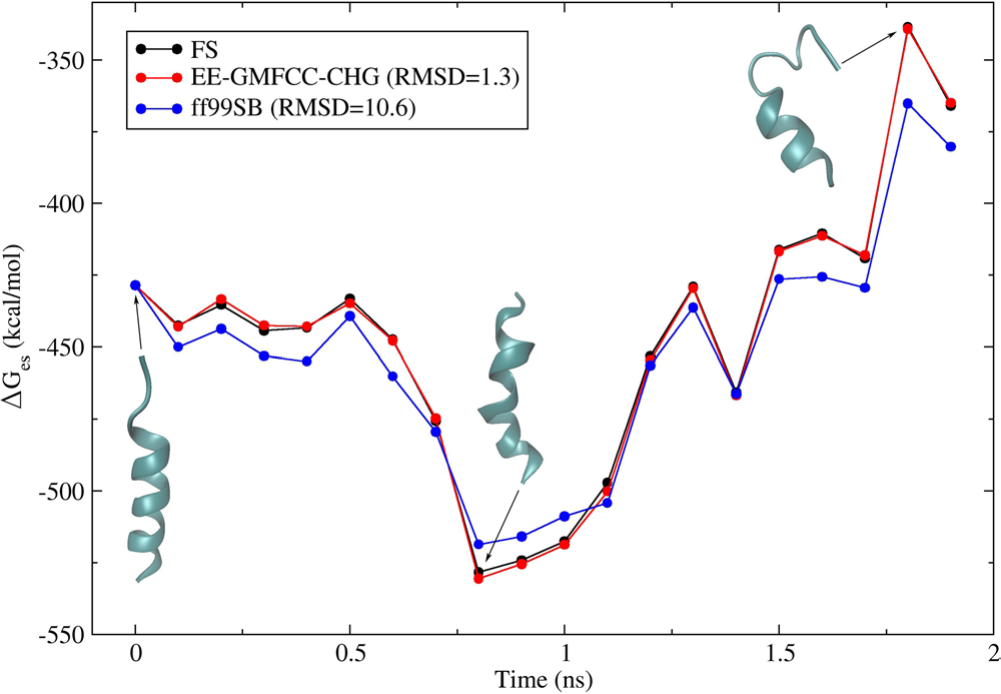
Comparison of the relative electrostatic solvation energies of 20 conformers selected from 2 ns MD simulation between the standard full system calculations (black) and ff99SB force field (blue), EE-GMFCC-CHG (red), respectively, for the proteins 2I9M. The QM calculations for fitting protein atomic charges are performed at the HF/6-31 g* level. The distance threshold λ was set to 4.0 Å. The electrostatic solvation energy of the first conformer calculated from FS QM calculations was taken as reference.

Most of biological processes occur in solution. The solvent effect plays important roles in mediating biological processes such as protein-ligand, protein-protein interaction and protein folding. During folding process, the conformation of a protein changes dramatically from random coil to its functional three-dimensional structure. The interplay between the protein and solvent seriously affects the pathway of protein folding. Accurate calculations of solvent energies of proteins are significant for revealing the roles of solvent effect in these biological processes. The relative electrostatic solvent energies of 20 different conformations of a real protein 2I9M are calculated by combining the EE-GMFCC-CHG with the PB implicit solvent model and compared with the calculated results using the FS *ab initio* calculations. The solvent effect is introduced by iteratively introduction of the induced surface charges as background charges in the QM calculations. The relative electrostatic solvation energies of 20 conformations of protein 2I9M are shown in Fig. 4. For comparison, the calculated results based on Amber99SB force field are also shown in Fig. 4. Protein 2I9M is a prototype of $\,{\rm{\alpha }}$-helix polypeptide which has been studied in previous theoretical protein folding works^6^. Our 2 ns molecular dynamic (MD) simulation of protein 2I9M with Amber99SB force field shows that its native conformation is not stable and unfolding occurs. The representative three-dimensional structures of three different conformations selected from the MD simulation are presented in Fig. 4. One can see that the conformation of the protein 2I9M changes dramatically in the 2 ns MD simulation. This is because the Amber99SB force field is considered to disfavor the $\,{\rm{\alpha }}$-helix structure. As a result, it is worth studying the free energy profile of protein 2I9M with more sophisticated methods. Figure 4 shows that the electrostatic solvation energies of the 20 different conformations undergo large fluctuation between −530 and −330 kcal/mol. The calculated electrostatic solvation energies with the EE-GMFCC-CHG are in excellent agreement with the FS calculations with RMSD of 1.3 kcal/mol, in contrast, the results obtained from Amber99SB force field show much larger deviations with RMSD of 10.6 kcal/mol which has about 1 order of magnitude larger than that of the EE-GMFCC-CHG. Furthermore, the deviation of calculated absolute electrostatic solvation energy between EE-GMFCC-CHG and FS QM calculations ranges from −0.3 to −5.5 kcal/mol, see Table S1 of the Supporting Information. While the deviation of the results based on Amber99SB force field from FS QM calculations ranges from 56 to 93 kcal/mol. In addition, the electrostatic solvation energies calculated by the EE-GMFCC-CHG are all lower than the FS results. The mean unsigned error (MUE) of the EE-GMFCC-CHG is 2.79 kcal/mol which is much smaller than that of Amber99SB force field. It clearly shows that the errors from Amber99SB force field calculations are significantly larger than those calculated by the EE-GMFCC-CHG. The comparison shows that the including quantum mechanical information is very important for predicting accurate electrostatic solvation energies of proteins.

**Figure 5:**
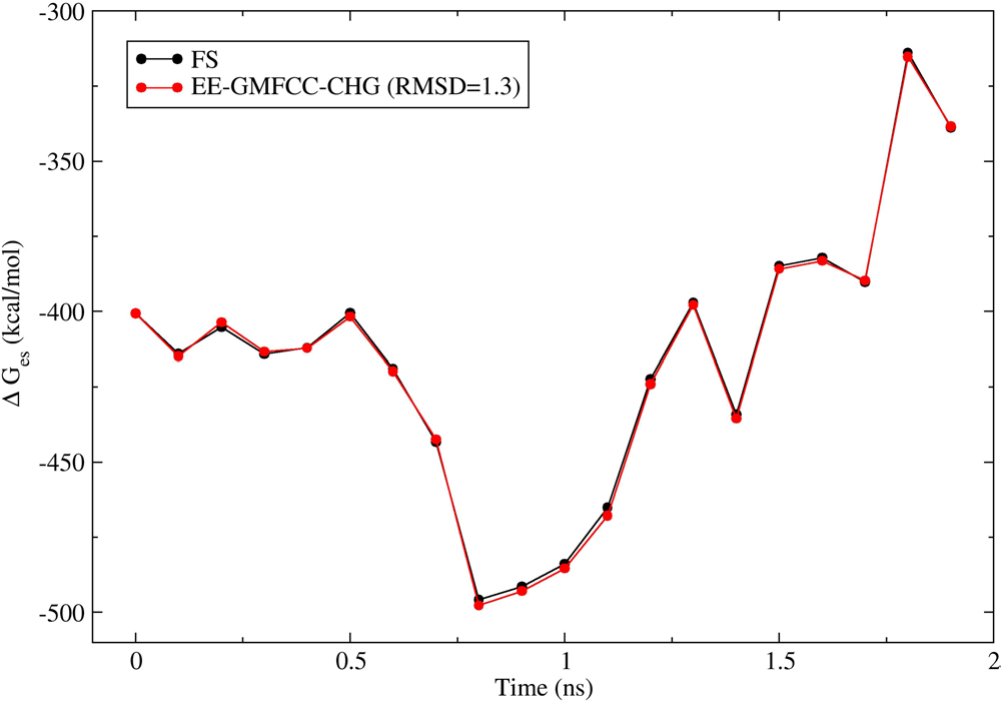
Similar to Fig. 4, but for M06-2×/6-31 G* calculations on 20 conformers of 2I9M.

To further demonstrate the capability of the EE-GMFCC-CHG in reproducing the solvation energy with DFT exchange-correlation functionals, the electrostatic solvation energies of the 20 different conformations of protein 2I9M are calculated using M06-2× method with the 6-31 G* basis set and shown in Fig. 5. One can see that the relative electrostatic energies evaluated using the EE-GMFCC-CHG agree well with that of the FS QM calculations with RMSD of 1.3 kcal/mol. The calculated electrostatic solvation energies of the 20 different conformations of protein 2I9M at M06-2×/6-31 G* level ranges from −310 to −500 kcal/mol which is a little higher than the results obtained at HF/6-31 G* level, see Table S2 of the Supporting Information. The deviation of calculated absolute electrostatic solvation energy between the EE-GMFCC-CHG and the FS QM calculations is also small which ranges from −0.3 to −4.7 kcal/mol. Similar to the results of the HF method, the electrostatic solvation energies calculated by the EE-GMFCC-CHG with DFT (M06-2×/6-31 G*) method are also all lower than the FS results. The results demonstrate that EE-GMFCC-CHG could reproduce the solvation energy of the protein well with both HF and DFT method.

## Conclusions

In this work, we developed a charge model termed EE-GMFCC-CHG for accurately modeling the molecular electrostatic potential of proteins. The EE-GMFCC-CHG is obtained by fitting the ESP calculated from accurate electronic structure calculation of protein. Therefore, it contains almost all quantum effects (the polarization and charge transfer effects) of a specific structure of a protein. The EE-GMFCC method is computationally efficient and linear-scaling. The individual QM calculations of all fragments can be carried out in parallel. In reproducing MEP of protein, EE-GMFCC-CHG gives an excellent agreement with full system *ab initio* QM method and shows a significant improvement relative to popular MM force field. By analysis of RMSDs (reference to the full system QM results) of the MEP calculated from the EE-GMFCC-CHG with different two-body distance threshold $\,{\rm{\lambda }}$, we have shown that accounting for the polarization and charge transfer effect over the sequently-neighboring residues is very important for accurately reproducing the *ab initio* QM electrostatic potential of proteins, following by the quantum mechanical two-body effects between the non-neighboring residues in close contact.

By combining the EE-GMFCC-CHG with the implicit water model based on the PB equation, we developed a quantum chemical method for modeling the solvation effect of proteins. With respect to popular MM force fields, the EE-GMFCC-CHG-PB method could take the polarization effect between solute and solvent into consideration by iteratively introducing protein surface induced charges in the fitting process and therefore it shows a significant improvement in the description of electrostatic solvation energetics of proteins. The error of EE-GMFCC-CHG in modeling relative/absolute electrostatic solvation energies of protein is very small with reference to the full system QM calculation. The EE-GMFCC-CHG-PB method will thus be a useful tool for modeling electrostatic solvation energetics of solvated proteins.

## Electronic supplementary material

Supplementary Information
