# Supplementary material for: A quantum mechanical computational method for modeling electrostatic and solvation effects of protein
Source: Sci Rep. 2018 Apr 3;8:5475. doi: 10.1038/s41598-018-23783-8 (PMC5882933; doi:10.1038/s41598-018-23783-8)
Supplement: Supplementary file 1 — Supplementary Information [file 41598_2018_23783_MOESM1_ESM.pdf]

Supplementary Information

A quantum mechanical computational method for modeling electrostatic and solvation effects of protein

Xianwei Wang[1], Yang Li[2], Ya Gao[3], Zejin Yang[1], Chenhui Lu[4], Tong Zhu[5,6*]*

[1] *College of Science, Zhejiang University of Technology, Hangzhou, Zhejiang 310023, China*

[2] *School of Information Science and Engineering, Shandong Agricultural University, Taian, 271018, China*

[3] *College of Fundamental Studies, Shanghai University of Engineering Science, Shanghai 201620, China*

[4] *College of Mechanical Engineering, Shanghai University of Engineering Science, Shanghai 201620, China*

[5] *College of Chemistry and Molecular Engineering, East China Normal University, Shanghai, 200062, China*

[6] *NYU-ECNU Center for Computational Chemistry at NYU Shanghai, Shanghai 200062, China*

[*] Corresponding author (wxw263@163.com, tzhu@lps.ecun.edu.cn).

Table S1. The absolute electrostatic solvation energies of 20 different conformations of protein 2I9M obtained from standard full system calculations and deviation from full system calculations of the results obtained from the EE-GMFCC-CHG and Amber99SB force field. The QM calculations are performed at HF/6-31G* level.

Conformation	Full system (kcal/mol)	Deviation from full system calculations (kcal/mol)	
		EE-GMFCC-CHG (kcal/mol)	Amber (kcal/mol)
1	-428.51	-2.38	83.42
2	-442.5	-2.87	75.86
3	-435.4	-0.38	75.11
4	-444.3	-0.61	74.6
5	-443.38	-1.88	71.8
6	-433.22	-4.02	77.35
7	-447.38	-2.77	70.62
8	-475.63	-1.47	79.59
9	-528.37	-4.62	93.09
10	-524.24	-3.65	91.73
11	-517.6	-3.61	92.1
12	-497.18	-5.43	76.34
13	-453.17	-3.87	80.22
14	-429.03	-2.91	76.14
15	-465.72	-3.49	82.81
16	-416.14	-2.93	73.16
17	-410.47	-3.23	68.26
18	-419.22	-1.24	73.2
19	-338.5	-3.08	56.74
20	-365.92	-1.45	69.02
MUE		2.79	77.06
RMSE		3.07	77.53

Table S2. Similar to Table S1, but for M06-2X/6-31G* calculations on 20 conformers of 2I9M.

Conformation	Full system (kcal/mol)	Deviation from full system calculation of EE-GMFCC-CHG (kcal/mol)
1	-400.66	-2.00
2	-414.08	-2.78
3	-405.28	-0.35
4	-414.22	-1.17
5	-412.13	-2.12
6	-400.52	-3.26
7	-418.98	-2.83
8	-443.23	-1.23
9	-495.80	-3.94
10	-491.45	-3.50
11	-483.91	-3.52
12	-465.12	-4.68
13	-422.41	-3.66
14	-397.13	-2.74
15	-434.19	-3.36
16	-384.85	-3.06
17	-382.14	-3.05
18	-390.21	-1.53
19	-313.86	-3.37
20	-338.74	-1.70
MUE		2.69
RMSE		2.89
